# Role of Natural Antioxidants from Functional Foods in Neurodegenerative and Metabolic Disorders

**DOI:** 10.1155/2018/1459753

**Published:** 2018-10-14

**Authors:** José C. T. Carvalho, Caio P. Fernandes, Julio B. Daleprane, Maria S. Alves, Didier Stien, N. P. Dhammika Nanayakkara

**Affiliations:** ^1^Amapá Federal University, Amapá, Brazil; ^2^Rio de Janeiro State University, Rio de Janeiro, Brazil; ^3^Juiz de Fora Federal University, Minas Gerais, Brazil; ^4^Sorbonne Universités, Paris, France; ^5^University of Mississippi, Oxford, USA

A food, despite its nutritional value, is defined as functional if it is appropriately shown to affect beneficially one or more target functions in the body, being a source of mental and physical well-being, and contributing to the prevention and reduction of risk factors of various diseases, or improving certain physiological functions [1]. Additionally, the ingestion of foods containing natural antioxidants such as fruits, herbs, legumes, oilseeds, whole grains, and vegetables, as well as the consumption of processed foods supplemented with natural antioxidants like vitamins C and E, carotenoids, and polyphenols, can provide the desired antioxidant status, contributing to the prevention of the neurodegenerative and metabolic disorders [2, 3]. In fact, it is noteworthy that the antioxidants play a crucial role in both food systems and in the human body to reduce oxidative processes and, consequently, neurodegenerative diseases and metabolic dysfunctions.

In this context, this special issue offers several articles reporting distinct approaches with results that corroborated the relevance of natural antioxidants in the minimization of degenerative disorders and metabolic dysfunctions. It contains six papers, and the details are presented below.

G. Li et al. investigated if the three polyphenol stilbenes (rhaponticin (RHAc), desoxyrhaponticin (dRHAc), and rhapontigenin (RHAg)) from Fenugreek (*Trigonella foenum-graecum* L.) seeds were able to improve the insulin sensitivity and mitochondrial function in 3T3-L1 adipocytes. These authors demonstrated that these compounds markedly improved the insulin sensitivity and mitochondrial function in 3T3-L1 adipocytes, RHAc being the most efficient among them.

K. S. Cho et al. reviewed the latest studies on the effects of carotenoids on neurodegenerative diseases in humans, the animal and cellular model investigations on the beneficial effects of these compounds on neurodegeneration, and the possible mechanisms and limitations of these compounds in the treatment and prevention of neurological diseases.

Novel pharmacological targets have been investigated for the treatment of diseases associated with oxidative processes and metabolic alterations by B. C. S. Santos et al. Original data revealed that methyl chavicol (MC) and its synthetic analogue 2-((4-methoxyphenyl)methyl) oxirane (MPMO) presented an antioxidant potential in trials that differ in relation to the evaluated mechanism, and MC was more effective than MPMO when the antilipase activity was evaluated, including by the molecular docking study.

G. P. F. Arrifano et al. analyzed the possible modulation of GABAergic homeostasis within synaptic clefts *in vitro* using clarified açaí (*Euterpe oleracea*) juice, to prevent seizures. According to these authors, *E. oleracea* can improve GABAergic neurotransmission via interactions with the GABA_A_ receptor and modulation of GABA uptake, possibly leading to the accumulation of endogenous GABA in the synaptic cleft and enhancing the inhibitory neurotransmission in the brain.

H. Zhao et al. described the antioxidant and hepatoprotective activities of hot-water-extractable polysaccharides (H-SMPS) and enzymatic-extractable polysaccharides (E-SMPS) isolated from spent mushroom substrates (SMS) of *Laetiporus sulphureus* in acute alcohol-induced mice. Their data showed that H-SMPS and E-SMPS have an antioxidant capability and potential hepatoprotective effects against alcohol-induced alcoholic liver disease.

C. G. de Souza et al. chemically studied the electroanalytical profile of the hydroalcoholic extract of “jabuticaba” (*Myrciaria cauliflora*) fruits (HEJ), their antioxidant capacity, and their effects on hypertensive animals after chronic treatment to associate the cardiovascular effects with the typical phytochemistry groups detected in this plant species. These authors observed that HEJ presents a high antioxidant potential, and the treatment with this hydroalcoholic extract attenuated the hypertension possibly improving the nitric oxide biodisponibility.

The Guest Editors hope that the readers of this special issue will find these readings attractive, delightsome, and mainly useful, making the update of this interesting and challenging subject easier than it is.

Finally, the Guest Editors are very grateful to the authors who submitted their precious research to this special issue, and they would also like to warmly acknowledge the reviewers for their excellent contribution to improve the quality of this work.
